# Tomonaga–Luttinger Spin Liquid and Kosterlitz–Thouless Transition in the Spin-1/2 Branched Chains: The Study of Topological Phase Transition

**DOI:** 10.3390/ma15124183

**Published:** 2022-06-13

**Authors:** Hamid Arian Zad, Azam Zoshki, Nerses Ananikian, Michal Jaščur

**Affiliations:** 1 Alikhanyan National Science Laboratory, Alikhanian Br. 2, Yerevan 0036, Armenia; ananik@yerphi.am; 2 Department of Theoretical Physics and Astrophysics, Faculty of Science, P. J. Sǎfárik University, Park Angelinum 9, 041 54 Kosice, Slovakia; zoshkia@yahoo.com (A.Z.); michal.jascur@upjs.sk (M.J.); 3 CANDLE Synchrotron Research Institute, Acharyan 31, Yerevan 0022, Armenia

**Keywords:** magnetization, intra-chain interaction, spin liquid phase, Kosterlitz–Thouless transition

## Abstract

In the present work, we provide a comprehensive numerical investigation of the magnetic properties and phase spectra of three types of spin-1/2 branched chains consisting of one, two and three side spins per unit block with intra-chain interaction and a uniform inter-chain interaction in the presence of an external magnetic field. In a specific magnetic field interval, the low-temperature magnetization of these chains shows a step-like behavior with a pronounced plateau depending on the strength and the type of intra-chain interaction being ferromagnetic or antiferromagnetic. We demonstrate that when inter-chain interaction $J_{1}$ is antiferromagnetic and intra-chain interaction $J_{2}$ is ferromagnetic, the magnetization of the models manifests a smooth increase without a plateau, which is evidence of the existence of a Luttinger-like spin liquid phase before reaching its saturation value. On the other hand, when $J_{1}$ is ferromagnetic and $J_{2}$ is antiferromagnetic, the low-temperature magnetization of the chain with two branches shows an intermediate plateau at one-half of the saturation magnetization that breaks a quantum spin liquid phase into two regions. The magnetization of the chain with three branches exhibits two intermediate plateaus and two regions of a quantum spin liquid. We demonstrate that the chains with more than one side spin illustrate in their ground-state phase diagram a Kosterlitz–Thouless transition from a gapful phase to a gapless spin liquid phase.

## 1. Introduction

Throughout the years, Heisenberg spin models have been considered as usual tools to characterize the destiny of a material in different circumstances [1,2,3,4,5,6,7]. For example, in references [8,9,10,11], the absence of long-range order was proved in the presence of an exchange interaction for 1-D and 2-D quantum Heisenberg systems. Magnetic properties of metals introduced in terms of spin models have attracted intense interest from researchers due to their potential implementations in condensed matter physics and materials science [12,13,14,15,16,17,18].

During the recent decades, substantial advances in designing single-chain magnets (SCMs) [19,20,21,22] have come to pass by means of 1-D quantum spin models. For the time being, heterometallic SCMs are important quantum ferrimagnetic chains for physical realizations of metal-containing polymers. They have already attracted a great deal of attention from chemists and physicists due to the fact that they exhibit a lot of unconventional magnetic properties at low temperatures. The main reason behind particular magnetic behavior is the interplay between ferromagnetic and antiferromagnetic spin states [23,24,25].

The magnetization of 1-D Heisenberg chain models reveals fascinating features such as an intermediate plateau [17,18,26,27,28,29,30,31,32,33,34,35,36], jumps and steps [37,38,39,40], and spin liquid states [24,25,41,42]. In recent works, the ground-state phase diagram and the low-temperature magnetization process of several spin-1/2 Heisenberg branched chains have been examined, whose magnetic structure is inspired by the heterobimetallic coordination polymers [43,44]. In reference [45], the ground-state phase transition and magnetic properties of a frustrated spin chain with side chains have been investigated in detail. Motivated by novel, highly correlated low-dimensional systems, V. O. Cheranovskii et al. studied the magnetization process of a series of spin-1/2 chains with different types of intra-chain interactions at low temperatures [46]. The Kondo-necklace models with similar spin structure to the branched chains were considered, and some important results for their ground-state phase transition have been reported [47,48].

Despite the fact that linear spin chains with side spins comprise a rich physics in materials science and technology, less attention has been paid to the effect of the number of branches, to the nature of interactions between chain and side spins, and to the effect of intra-chain interactions on the ground-state phase spectra of these models. In the present paper, we will numerically discuss the low-temperature magnetic properties of the spin-1/2 XXX Heisenberg model on the three different branched chains with one, two and three intra-chain interactions in their unit blocks. Hereafter, we label these chains as chain $\Upsilon_{1}$, chain $\Upsilon_{2}$ and chain $\Upsilon_{3}$, respectively. In fact, we will widely discuss the problem concerning the effects of side spins and their exchange interaction on the ground-state phase spectra and the magnetization process of the above-described spin-1/2 Heisenberg branched chains. To gain a reasonable insight into the low-temperature magnetization process of the chain models, we use Quantum Monte Carlo (QMC) simulations under the subroutine dirloop–sse–a package from the Algorithms and Libraries for Physics Simulations (ALPS) project, which prepares a full generic implementation of the QMC simulations for spin lattices [49,50].

The paper is organized as follows. In Section 2, the spin-1/2 XXX Heisenberg model on the three different types of 1-D branched chains with one, two and three intra-chain interactions per unit cell is introduced. In Section 3, we numerically investigate the magnetic properties of the introduced spin chains by implementing QMC simulations. The following input parameters are considered for the applied sse loop: $1\times 10^{5}$ thermalization, $1\times 10^{4}$ MC sweeps, assuming periodic boundary conditions. Next, we report the most interesting results obtained for the magnetic behavior of the spin chains $\Upsilon_{1}$,$\Upsilon_{2}$ and $\Upsilon_{3}$ under two different conditions considered for the nature of inter- and intra-chain interactions $J_{1}$ and $J_{2}$. In particular, the effect of the intra-chain interaction $J_{2}$ between side spins and chains on the low-temperature magnetization of the introduced models will be discussed in detail. The conclusions are drawn in Section 4.

## 2. The Model

**Figure 1:**
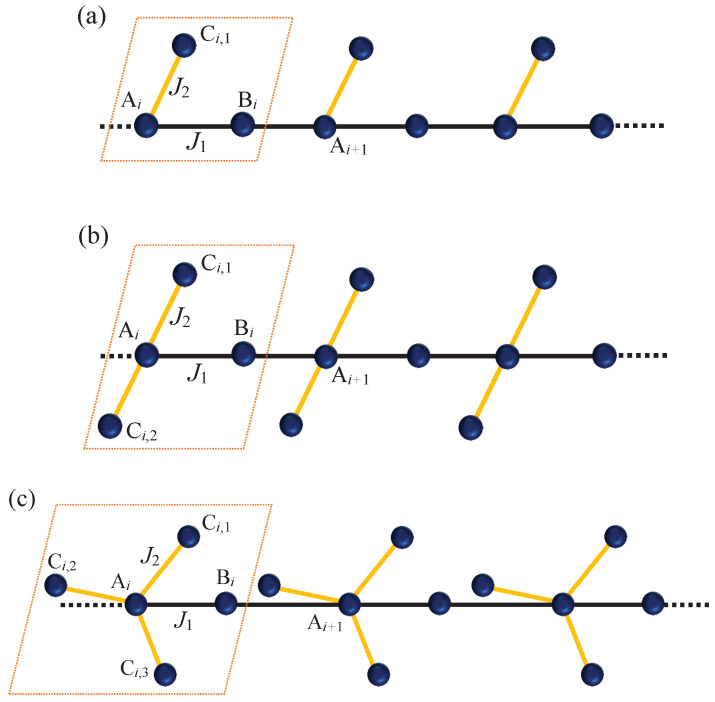
Schematic structure of the spin-1/2 XXX Heisenberg model on the branched chains (**a**) $\Upsilon_{1}$ with one, (**b**) $\Upsilon_{2}$ with two, and (**c**) $\Upsilon_{3}$ with three intra-chain interactions $J_{2}$. $J_{1}$ denotes inter-chain interaction. In each case, the dotted rectangle indicates a unit cell that uniformly repeats throughout the chains. Solid balls labeled as A, B, and C represent spin-1/2 particles in a unit block, and the balls marked with $C_{i}$ indicate the side spins.

Let us consider the spin-1/2 XXX Heisenberg model on the branched chains illustrated in Figure 1 described by an effective Hamiltonian

(1) $$\begin{array}{c} H=\sum_{i=1}^{N}\left\{J_{1}\left(S_{A_{i}}\cdot S_{B_{i}}+S_{B_{i}}\cdot S_{A_{i+1}}\right)+\sum_{j=1}^{n}\left[J_{2}\left(S_{A_{i}}\cdot S_{C_{i,j}}\right)-g\mu_{B}H\left(S_{A_{i}}^{z}+S_{B_{i}}^{z}+S_{C_{i,j}}^{z}\right)\right]\right\} \end{array}$$

where *N* is the number of unit cells, and *n* accounts for the number of side spins. The total number of spins L=(n+2)N is considered for the systems under periodic boundary conditions, where $S_{A_{N+1}}\equiv S_{A_{1}}$. $J_{1}$ and $J_{2}$ are, respectively, inter- and intra-chain exchange interactions between each of the two nearest neighbor spins, *H* is the applied magnetic field in the *z*–direction. *g* and $\mu_{B}$ are g-factor and Bohr magneton, respectively. The XXX interaction between each pair of spins can be formulated as

(2) $$\begin{array}{c} S_{\alpha }\cdot S_{\alpha^{\prime }}=S_{\alpha }^{x}S_{\alpha^{\prime }}^{x}+S_{\alpha }^{y}S_{\alpha^{\prime }}^{y}+S_{\alpha }^{z}S_{\alpha^{\prime }}^{z} \end{array}$$

in which spin-1/2 operators are given by (ℏ=1)

(3) $$\begin{array}{c} s^{x}=\frac{1}{2}\left(\begin{array}{cc} 0 & 1\\ 1 & 0 \end{array}\right),\;s^{y}=\frac{1}{2}\left(\begin{array}{cc} 0 & -i\\ i & 0 \end{array}\right),\;s^{z}=\frac{1}{2}\left(\begin{array}{cc} 1 & 0\\ 0 & -1 \end{array}\right) \end{array}$$

Henceforward, to have a dimensionless parameter space, in our numerical tasks, the intra-chain exchange interaction $J_{2}$ will be considered as the energy unit.

## 3. Magnetic Properties

To demonstrate the magnetic properties of the three types of 1-D spin-1/2 Heisenberg branched chains $\Upsilon_{1-3}$, we have calculated the low-temperature magnetization process using the QMC method with the stochastic series expansion implementation from the ALPS package with L=120 number of spins. Two following conditions are supposed for the exchange couplings $J_{1}$ and $J_{2}$:

❖ Condition (I): We assume ferromagnetic coupling $|J_{2}|$ is the energy unit ($J_{2}<0$) where the magnetization of the models is examined for different antiferromagnetic interaction ratios $J_{1}/\left|J_{2}\right|>0$.
❖ Condition (II): We assume antiferromagnetic coupling $J_{2}>0$ is the energy unit, and the magnetization is investigated for various fixed values of the ferromagnetic interaction ratio $J_{1}/J_{2}<0$.

The main goal of the above selection of antiferromagnetic–ferromagnetic interactions is to study the competition between antiferromagnetism and ferromagnetism to determine the predomination of the gapless spin liquid phases and to detect the Kosterlitz–Thouless (KT) critical point in the ground-state phase spectra of the models.

### 3.1. The Heisenberg Spin-1/2 Branched Chains under Condition (I)

**Figure 2:**
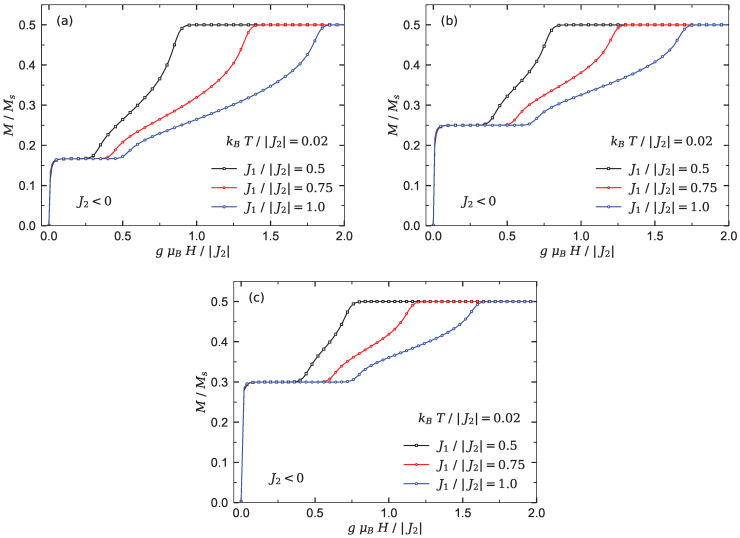
QMC results obtained for the magnetization curve of the three Heisenberg branched chains at low temperatures $k_{B}T/\left|J_{2}\right|=0.02$ under the condition (I) where three different fixed values of the inter-chain interaction $J_{1}/\left|J_{2}\right|=\left\{0.5,0.75,1.0\right\}$ are considered. (**a**) The magnetization of chain $\Upsilon_{1}$. (**b**) The magnetization of chain $\Upsilon_{2}$. (**c**) The magnetization of chain $\Upsilon_{3}$.

Figure 2 displays QMC simulations of the magnetization per saturation value $M/M_{s}$ for the chains $\Upsilon_{1-3}$ versus the magnetic field at low enough temperature $k_{B}T/\left|J_{2}\right|=0.02$ (*T* is the temperature and $k_{B}$ is the Boltzmann constant).

As can be seen in Figure 2, the magnetization of each spin chain shows an abrupt jump when the magnetic field increases from zero. This quantity reaches an intermediate magnetization plateau at the fractional value $M/M_{s}=n/\left(n+2\right)$. This single plateau coincides with the gapful Lieb–Mattis (LM) ferrimagnetic ground state [25,51]. Obviously, increasing the number of side spins *n* results in enhancing the height and width of LM plateau. The intermediate plateau terminates at a critical magnetic field due to a quantum phase transition to a Luttinger spin liquid phase. Evidently, increasing the interaction ratio $J_{1}/\left|J_{2}\right|$ enhances the region of the spin liquid phase, as well as the saturation field, while increasing the number of side spins restricts the region of the spin liquid phase and decreases the saturation field (compare Figure 2a with Figure 2b,c).

### 3.2. The Heisenberg Spin-1/2 Branched Chains under Condition (II)

**Figure 3:**
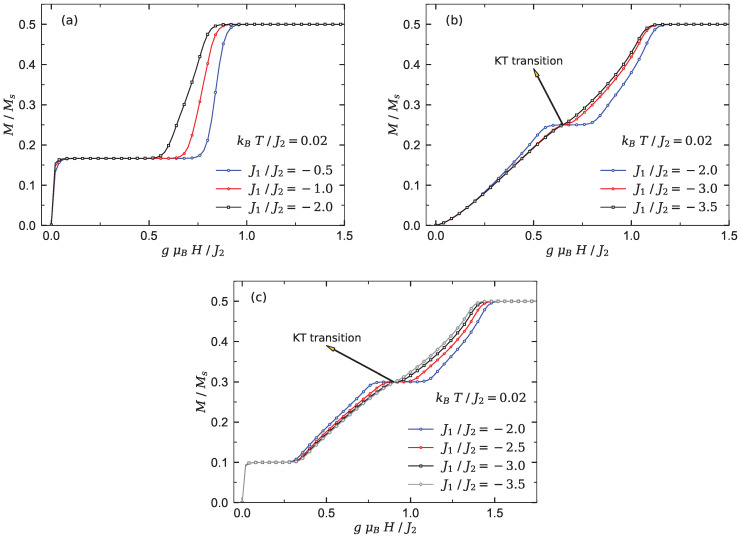
QMC results obtained for the magnetization curve of the three Heisenberg branched chains at low temperatures $k_{B}T/J_{2}=0.02$ under the condition (II), assuming a few fixed values of the inter-chain interaction $J_{1}/J_{2}$. (**a**) Chain $\Upsilon_{1}$. (**b**) Chain $\Upsilon_{2}$. (**c**) Chain $\Upsilon_{3}$.

The spin chains $\Upsilon_{2}$ and $\Upsilon_{3}$ show more interesting magnetic properties under the condition (II). To shed light on this issue, we plot the magnetization curve of models $\Upsilon_{1-3}$ versus the magnetic field at $k_{B}T/J_{2}=0.02$, where a few selected values of the interaction ratio $J_{1}/J_{2}$ are supposed. The model $\Upsilon_{1}$ displays in its magnetization process a single intermediate plateau at one-third of the saturation magnetization (see Figure 3a). When the interaction ratio $J_{1}/J_{2}$ becomes ferromagnetically stronger, a spin liquid phase emerges into the ground-state phase spectra of this model. 

By inspecting Figure 3b, we realize that except for the case shown in Figure 2b, the magnetization of the model $\Upsilon_{2}$ under condition (II) shows a wide spin liquid phase at low magnetic fields instead of the LM ferrimagnetic phase. An intermediate magnetization plateau at one-half of the saturation value appears at moderate magnetic fields. We find that this intermediate magnetization plateau terminates at a quantum KT critical point when the absolute value of interaction ratio $J_{1}/J_{2}$ increases.

**Figure 4:**
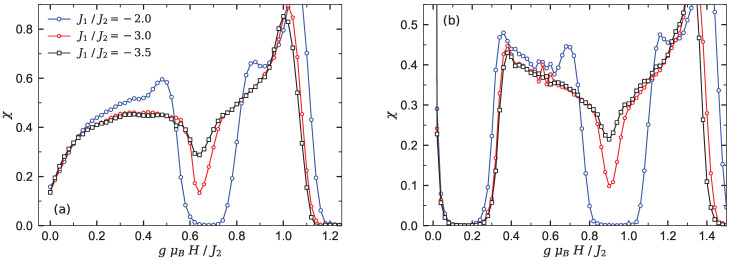
The low-temperature susceptibility χ versus magnetic field for (**a**) the Heisenberg branched chain $\Upsilon_{2}$ and (**b**) chain $\Upsilon_{3}$, at $k_{B}T/J_{2}=0.02$ under the condition (II). Three different values of the inter-chain interaction $J_{1}/J_{2}=\left\{-2.0,-3.0,-3.5\right\}$ are considered for the both panels.

The sharp decrease in the susceptibility χ of chain $\Upsilon_{2}$ (see Figure 4a) nearby the relevant quantum critical point $g\mu_{B}H/J_{2}\approx 0.65$ is solid evidence of the KT transition.

When the number of side spins increases, the branched chain illustrates different magnetization behavior at low temperature. For instance, in Figure 3c, the magnetization of chain $\Upsilon_{3}$ is presented for different fixed values of the interaction ratio $J_{1}/J_{2}$. It is quite obvious that the magnetization of model $\Upsilon_{3}$ manifests two intermediate plateaus at one-fifth and three-fifth of the saturation magnetization. In fact, by increasing the magnetic field from zero, the magnetization abruptly jumps to the first intermediate plateau. This quantity starts to increase smoothly from the first plateau at a critical magnetic field ($g\mu_{B}H/J_{2}\approx 0.3$) and reaches the second intermediate plateau, and successively a second smooth increase of the magnetization starts from another critical magnetic field nearby $g\mu_{B}H/J_{2}\approx 1.1$. These smooth increases of the magnetization are reminiscent of the existence of the Tomonaga–Luttinger spin liquid in the ground-state phase diagram of chain $\Upsilon_{3}$. It can be seen from Figure 3c that the second intermediate plateau ($M/M_{s}=3/5$) appeared in the magnetization curve of the branched chain $\Upsilon_{3}$ monotonically shrinks upon increasing the interaction ratio $J_{1}/J_{2}$. This plateau eventually disappears at a quantum critical KT point $g\mu_{B}H/J_{2}\approx 0.9$. The steep decrease in the susceptibility χ shown in Figure 4b close to this point denotes a quantum KT transition. Clearly, magnetic susceptibility χ vanishes for the gapful phases, while it has unconventional behavior with nonzero value within the gapless spin liquid phase regions.

## 4. Conclusions

To summarize, we have numerically investigated the magnetic properties of the spin-1/2 XXX Heisenberg model on the three different 1-D branched chains consisting of one, two and three intra-chain interactions per unit cell. Indeed, we have examined the magnetization and magnetic susceptibility of these spin systems by employing the QMC method.

When the intra-chain interaction is ferromagnetic and inter-chain interaction is antiferromagnetic, the magnetization of all chains shows a single intermediate plateau whose height is related to the number of side spins. This plateau terminates at a critical field, and successively, a gapless Tomonaga–Luttinger liquid phase arises, where the magnetization starts to increase smoothly until it reaches its saturation value. We understood that by increasing the number of side spins, the phase region corresponds to the Luttinger liquid phase diminishes.

On the other hand, when the intra-chain interaction is antiferromagnetic and inter-chain interaction is ferromagnetic, the low-temperature magnetization of the branched chains with more than one side spin per unit cell exhibit more rich magnetic properties. Under this situation, the magnetization of the branched chain with two side spins shows an intermediate plateau at one-half of saturation value that breaks the quantum spin liquid into two areas. We have concluded that for a relatively strong value of the ferromagnetic inter-chain interaction this plateau terminates at a quantum KT critical point.

Moreover, we have observed that the magnetization of chain with three side spins shows two intermediate plateaus at one-fifth and three-fifth of the saturation magnetization. Another interesting finding from our investigations is that with an increase in the inter-chain interaction, the intermediate plateau at three-fifth of saturation magnetization gradually shrinks and eventually ends up at a KT point, while the intermediate plateau at one-fifth of saturation magnetization becomes more robust.

We theoretically concluded that the considered Heisenberg branched chains reveal an interesting magnetic response to the quantity and quality of the intra-chain interaction. It is expected that our results could be useful for explaining the magnetic behavior of various metal-containing polymers with different side spins.
